# Easy Prestressing of FRP for Strengthening RC Beams: Experimental Study with an Analytical Approach

**DOI:** 10.3390/polym17121628

**Published:** 2025-06-12

**Authors:** Gokhan Sakar, Huseyin Kursat Celik

**Affiliations:** 1 Department of Civil Engineering, Dokuz Eylul University, İzmir 35210, Türkiye; 2 Department of Civil Engineering, Izmir University of Economics, Balçova, İzmir 35330, Türkiye; kursat.celik@ieu.edu.tr

**Keywords:** fiber-reinforced polymers, prestress, easy prestressing machine, section analysis, moment-curvature

## Abstract

This study investigates strengthening reinforced concrete (RC) beams using fiber-reinforced polymers (FRPs). Nine samples were cast and strengthened with varying parameters, including the width, number of laminates, use of anchors, and application of prestressing. A novel device—the easy prestressing machine (EPM)—was developed to apply prestress. The EPM is lightweight and operable manually, enabling up to 10% prestressing. All specimens were tested under three-point bending until failure, and load-displacement curves were recorded. An analytical method based on curvature increment and incorporating material nonlinearities is also proposed to estimate the load-displacement response of RC beams with and without FRP strengthening. Both experimental and analytical results are presented and compared. The analytical model strongly agreed with the experimental results, showing Pearson correlation coefficients exceeding 90% for most specimens. According to the experimental findings, applying FRP, particularly when combined with anchorage and prestressing, increased the load-bearing capacity by up to 45%. Anchorage and prestressing effectively mitigate premature debonding, with prestressing showing a more pronounced impact on enhancing bond performance and load capacity. Based on the results, conclusions regarding the analytical model, structural behavior, and optimal strengthening strategies are discussed.

## 1. Introduction

Undoubtedly, one of the most common strengthening methods used in recent years is fiber-reinforced polymer (FRP) reinforcement to improve the mechanical properties of reinforced concrete (RC) members. In recent decades, FRP strengthening methods and techniques have rapidly developed to repair, rehabilitate, and strengthen damaged or undamaged RC structures. These methods address various aspects, such as bending, shear, etc. [1,2,3,4,5,6].

Being a favorable option for this purpose can be explained by the fact that FRP is a lightweight material with high tensile strength. It can be easily and effectively applied to a RC element with epoxy resin to provide significant strengthening in bending. FRP strengthening can vastly improve the flexural capacity of a RC member without enlarging the cross-section. Thus, FRP is becoming increasingly popular as a strengthening material [7,8,9]. Despite this, there are some drawbacks to FRP applications. The interfacial interactions between FRP and concrete can lead to the premature failure of FRP plates, known as debonding [10]. Debonding refers to the bond failure between the FRP layer and the concrete surface. There are three main modes of debonding in RC elements: intermediate crack debonding, concrete cover separation, and plate-end interfacial debonding. In short, debonding can occur either in the FRP layer’s span or end region. The strengthened element will deform under bending as the load increases. When the shear stress between the FRP and concrete layer exceeds the limits, debonding occurs before the fracture of the FRP. Debonding is undesirable due to its brittle nature and reduces the effectiveness of FRP strengthening [11,12,13].

Anchorages are one of the most effective methods for overcoming debonding. Using anchors, the slip between the FRP and the concrete surface is restrained, preventing debonding [14,15]. Several effective anchoring methods exist in the literature, such as using U-strips at the end of the section. Each method has advantages and disadvantages regarding application efficiency and complexity [16]. However, the use of anchors does not guarantee the prevention of debonding. There is still a risk of debonding due to interface slip, cracks in the concrete, or semi-composite action, as debonding can occur along the entire span. Therefore, further solutions are needed to prevent debonding. Prestressing the FRP could be a viable solution [11].

Prestressing is a process in which the FRP strip is stressed before the beam is loaded. Prestressing FRP laminates generates prestress across the entire section, which can result in initial cambering. Consequently, the concrete is subjected to initial compression. When the beam is subjected to loading, the tension cracking of the concrete layer is delayed, and the initial stiffness of the beam increases [17]. Therefore, prestressed FRP applications can improve the strength properties of the beam. By completely closing existing cracks and delaying the formation of new ones, prestressed FRP laminates provide excellent crack control. Adding FRP force couples allows for higher loads and affects the stress distribution between the steel rebar and FRP plates. This reduces rebar stress under the same load as in a non-prestressed-strengthened beam [18,19].

Although prestressing is a desirable process, it is not an easy application. Mechanical anchorage and heavy equipment are required for this job. Further, using a mechanical anchorage may not always be feasible for several reasons. Mechanical anchors may damage the existing low-strength or damaged concrete elements [20]. Additionally, it is a time-consuming process, and the prestress level is often limited due to the strength of the concrete in the existing element. Another obstacle is prestressing equipment, which is generally performed with a hydraulic jack. The jack is difficult to transport to higher ground, and a structural framework is required to apply tension. A lightweight prestressing method may be a solution to this issue. For this purpose, thermal activation of shape memory alloys [21] and adopting the creep behavior of adhesives [22] may be highlighted. However, these methods may be seen as expensive and hard to control. Thus, another easy prestressing method may be the answer, and it was studied before by the corresponding author, which is called the easy prestressing machine (EPM) [23]. The design of this equipment was inspired by the observations made in previous studies. Applying a prestress is more critical than the prestress level. Therefore, using a high amount of prestress is not an obligation. From this perspective, low amounts of prestress can be applied by manpower.

**Figure 1:**
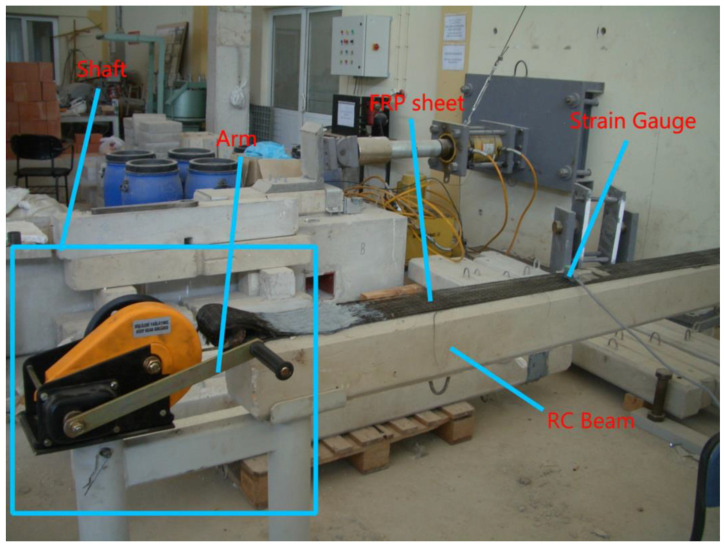
Easy prestressing machine (EPM).

The EPM comprises a shaft and an arm that can be pulled by the strength of the human arm. Wrapping the FRP sheet around the shaft is necessary to apply prestress to the FRP. Subsequently, the arm is rotated manually to stretch the FRP. The machine is simple and easy to use, and it may apply strains exceeding 0.002 mm/mm to the FRP (which corresponds to 450 MPa and 10% of the ultimate strain of FRP in this study). Further, the EPM is movable in both directions to avoid forming bubbles or voids while bonding the prestressed FRP. It should be noted that the success of the prestressing procedure depends on the bond at the interface. Additional measures by strain gauges were taken to ensure that a perfect bond was formed during the prestress period. Measurements from the strain gauge were used to control the application of prestress in terms of strain. In Figure 1, the EPM is illustrated.

First, the EPM is mounted in an appropriate position. The surface preparation of the concrete is done, and the FRP laminate is positioned to strengthen the face of the element. One side of FRP is anchored to the beam with a U-anchor, while the other side is connected to the EPM. Strain gauges are placed into FRP to measure the prestress level. By rotating the arm of the EPM, the FRP laminate will get tensioned. After reaching the required prestress level, epoxy is applied. After curing, the EPM connected the sides of the FRP laminates and anchored them too. Following that, the EPM is removed.

The disadvantages of the EPM should also be mentioned. The disadvantage of the EPM is that it applies prestress with manpower. A high amount of prestress may not be valid for an arm. Thus, using the EPM for high beams, bridges, etc., may be inappropriate due to limited force.

The present study investigates the strengthening of RC beams using prestressed FRP laminates. The effectiveness of prestressing, with and without anchorage, is evaluated through experimental testing. The key experimental findings are critically analyzed and discussed. An analytical approach was developed based on ACI 440.2R-22 [24] and existing literature on the sectional analysis of FRP-strengthened RC elements. The relationships between load and displacement, observed failure modes, and the overall efficiency of prestressing are examined. Finally, conclusions are presented regarding the role of prestressing and the validation of the proposed analytical method.

## 2. Experimental Studies

**Figure 2:**
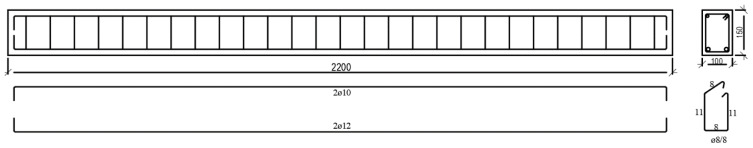
Beam geometry.

In this study, nine RC beams were cast, and experimental studies were carried out on several strengthening configurations. The test elements were reinforced concrete beams with 100 × 150 mm cross-sections. Each beam was reinforced with two 10 mm diameter top bars and two 12 mm diameter bottom bars. The beams were also confined with 8 mm stirrups to prevent shear failure. The yield strength of the reinforcing bars was determined to be 410 MPa. Unidirectional carbon FRP was used, with an elastic modulus of 231 GPa and a tensile strength of 4100 MPa. The thickness of the FRP strip was 0.12 mm. To determine the concrete strength of samples, cylindrical specimens were tested, and the 28-day compressive strength was found to be 20 MPa. Beam reinforcement details are shown in Figure 2. Subsequently, eight strengthened beam samples were fabricated with different strengthening configurations. In this study, the variables considered included FRP strip width, the number of layers, the presence of anchors, and the application of prestress. First, strips with widths of 50 mm and 100 mm were applied without anchors or prestress. Next, two 50 mm and 100 mm wide strip layers were used with an anchor to prevent premature debonding. Then, one layer of 50 mm and 100 mm wide FRP strips were applied with an anchor and 0.001 mm/mm pretension. Finally, two layers of 50 mm and 100 mm wide FRP strips were applied with an anchor and prestress. The U-strip was used for anchoring. The details of all samples are provided in Table 1. For notation, “RB” denotes the reference beam, and “SB” refers to the strengthened beam. The numbers 5 and 10 represent the FRP strip widths in centimeters, while “x2” indicates two layers of FRP. Finally, “A” denotes the use of a U-anchor, and “P” signifies the application of prestressing.

The total length of the beam was 2200 mm, and the loading span was 2000 mm. Using a hydraulic jack, the load was applied at the midspan, and strain gauges were attached to the midspan to monitor displacement. All samples were loaded until failure, and the results are given in Section 4.

To apply the FRP, the concrete surface was first roughened to enhance bonding between the FRP and the concrete. The surface was cleaned using air, and epoxy resin was applied to the surface for bonding with the FRP. The FRP strip was initially placed on the beam for prestressed samples, with one end anchored. Subsequently, the FRP strip was prestressed using the EPM, as shown in Figure 1. Prestressing was carried out manually by a single person’s arm, and the amount of prestress was monitored using a strain gauge, which measured strain. Once the 0.001 mm/mm strain was achieved, epoxy resin was applied to the FRP. After the resin had cured, the EPM was removed, and the beam was left to cure for 2 weeks.

## 3. Analytical Studies

Analytical solutions are a powerful tool for determining the behavior of a RC element in bending. Generally, sectional analysis with full interaction between concrete, rebar, and the FRP component is commonly used and is adopted in ACI 440 and other codes. According to the sectional analysis, the load capacity, stress–strain distribution, and load-displacement behavior can be determined based on the RC element’s failure mode. However, this method does not precisely capture the total behavior for several reasons. Material nonlinearities are often disregarded. Failure modes are generally considered solid limits, and load capacity and corresponding displacement are calculated at a specific point, rather than as a continuous curve. Thus, several authors have proposed a nonlinear section analysis, demonstrating a relatively high correlation with test results. However, failure modes are still predetermined [19,25,26,27]. To overcome this, several authors adopted an incremental method of moment-curvature analysis. This method obtains the load-displacement curve by calculating every load and displacement result for each curvature increment. Al-Zaid et al. [28] proposed a study to derive the full load-displacement curve using the curvature incremental method, assuming the fully composite behavior of the RC material. Further studies have considered the semi-composite action of the material and FRP–concrete interaction over the entire length of the element. Several works have been proposed using these methods [29,30,31,32,33]. Although this method successfully obtained the load-displacement curve, it is complex to generate, and the results are highly dependent on the bond-slip behavior of the FRP–concrete interface.

In conclusion, all methods proposed in the literature have some advantages and limitations. Based on the literature review, the authors propose a new approach for determining the load-displacement behavior of FRP-strengthened RC beams.

### Proposed Approach

**Figure 3:**
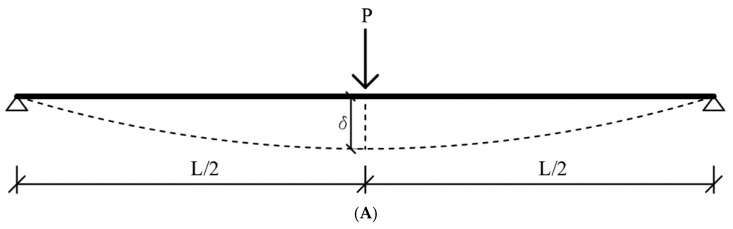

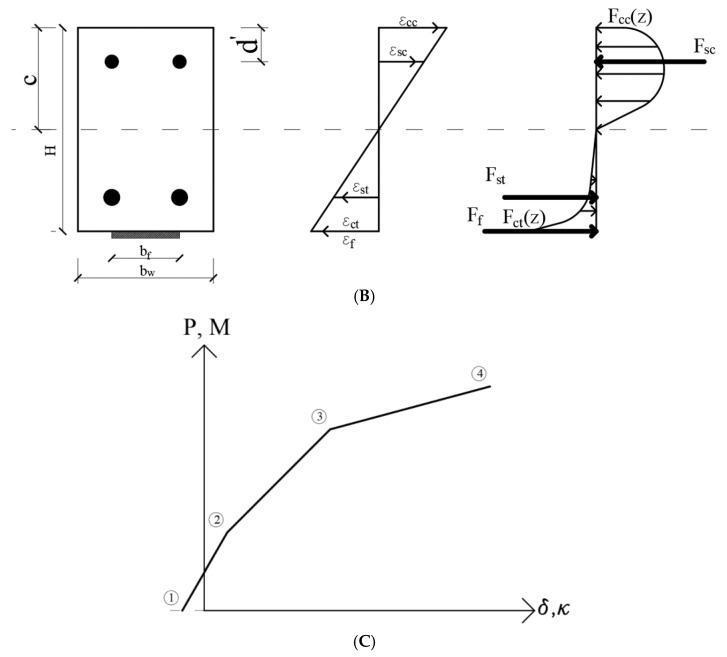
Behavior of RC beam in bending, with or without strengthening. (**A**) Loading scheme. (**B**) Stress and strain distribution of the section. (**C**) Characteristic of load-displacement curve.

Figure 3 shows the loading scheme, stress–strain relationship of the section, and representative load-displacement curve of the RC beam strengthened with prestressed FRP. It can be observed from the figure that a total of four actions occur when loading the RC beam, both with and without strengthening. In the first step, due to the prestressing of FRP, an initial cambering occurs in the beam in negative curvature. Thus, the displacement will start from a negative value when the loading begins. These results indicate compression in the RC section to compensate for the pretension. As the load increases, the cambering is switched from negative to positive. This means that the tensile stress will increase at the bottom of the section. Hence, the beam will reach a second stage, where tension cracking occurs. The concrete in the tension region will crack, causing a sudden loss of stiffness. If the beam is under-reinforced or the FRP ratio is sufficient to ensure rebar yielding, it will reach its yield point at the third stage. At this point, the stiffness of the beam is significantly decreased. Nevertheless, the beam can still support higher loads due to the presence of FRP. As the load increases, a fourth stage is reached if FRP debonding does not occur and the concrete does not crush. At this stage, the FRP loses its load-bearing capacity, and the beam will experience a sudden collapse. While this is a symbolic illustration, it is crucial for determining the stress–strain relationships of the beam in bending, both with and without FRP. Without FRP, rebar yielding leads to a yielding plateau, and once the concrete crushes, the beam fails. Without pretension, the load-displacement curve will start from zero, as no initial cambering occurs. Conversely, pretension increases the initial stiffness of the beam. In this study, the increment of curvature starts from a positive value because the initial strain is relatively low due to the low amount of prestressing applied.

Based on this expression, five actions occur within the section: concrete in compression, concrete in tension, top bar in compression, bottom bar in tension, and FRP in tension, with or without prestress. Since negative cambering can be neglected, prestress can be considered an initial strain, essentially, the limiting FRP strain. The general stress–strain relationship must be defined for each case to construct a continuous load-displacement curve for each scenario (non-strengthened, strengthened, and prestressed). Given the perfect composite assumption, this relationship should remain valid throughout the loading process.

In the proposed approach, several assumptions were made. The primary goal is to capture the complete behavior of the load-displacement curve from the initial crack to failure (see Figure 3). In this context, material nonlinearities and FRP debonding strain are crucial factors. To accurately capture the behavior of the RC beam in bending, monitoring the load-displacement data step by step is more effective than focusing solely on failure modes. The partial interaction of materials, or semi-composite action, can be disregarded because the behavior is primarily influenced by concrete tension softening. During the experimental study, concrete cracking happened prematurely before failing in the concrete–FRP interface. The slip between concrete and FRP did not happen in experiments. Therefore, the general beam bending theory, or Euler beam theory, is applicable. As a result, the linear strain distribution is maintained throughout the loading process. This approach can determine the load-displacement relationship through section analysis at midspan, where the highest strain and stress occur due to loading. Since FRP debonding can be expressed in terms of strain, intermediate crack debonding can be directly captured from the midspan curvature, and other debonding modes can also be defined based on midspan deformation.

A continuous moment-curvature function is required to obtain a continuous load-displacement curve. The moment-curvature relationship is given in Equation (1), valid throughout the loading history. Prior to analysis, concrete compressive strain (*εcc*), neutral axis depth (*c*), and curvature (*κ*) are unknown. However, the number of unknowns can be reduced by expressing the concrete strain in curvature and neutral axis depth. All strains in the section can be defined using strain compatibility, as shown in Equation (2). Once the strains are defined based on neutral axis depth and curvature, material stresses can be determined using the corresponding material constitutive laws (see Equation (3)). Before proceeding, strain should be defined using a parameter representing the distance through the section height to avoid computational errors. Forces acting on the section can be determined by integrating the stress function over the relevant height interval between the neutral axis depth and the section dimension, as given in Equation (4).

The force equilibrium must be satisfied. However, both curvature and neutral axis depth are unknown. The total forces acting on the section must be determined to ensure force equilibrium, as shown in Equation (5). The analytical method can then be solved using either analytical or numerical methods. Since the problem is iterative (due to curvature increments), only the neutral axis depth remains unknown, which can be found using a numerical iteration. In this study, the Newton–Raphson iteration method is used and presented in Equation (6). Once the neutral axis depth is determined, the moments of the sectional forces can be calculated using Equation (7).

With the moments known, the corresponding load at that curvature can be determined using beam theory (see Equation (8)). It is important to note that the problem considered in this study involves three-point bending, so the formulas provided are specific to three-point bending. To calculate displacement, the second moment of inertia must be known. As illustrated in Figure 3, the stiffness of a RC beam in bending changes during loading due to tension cracking in concrete. Therefore, the inertia is not constant. Instead, the effective moment of inertia (*I_eff_*) must be considered, which can be determined once the moment of that curvature is known (as shown in Equation (1)). After calculating the force and I_eff_, the displacement at that curvature can be found using Equation (10).

Equations (1)–(9) for each curvature increment can be obtained by applying the corresponding load and displacement data. A continuous load-displacement curve can be generated as curvature increases up to failure. Furthermore, if the material constitutive law is constructed as a continuous function, there is no need to assume a specific failure mode, such as rebar yielding or not. Several points should be addressed here: First, starting assumptions for the neutral axis depth (*ci*) values are required for the initial iteration. Since the neutral axis depth can vary along the section height, an initial assumption of *h*/2 can be used. For each subsequent curvature increment, the previous ci values can serve as starting values because increasing curvature results in a decreasing neutral axis depth.

Another consideration is inputting the initial and final curvatures for the analytical approach during the iteration. In beam bending theory, beam curvature can theoretically vary from 0 to infinity; however, this is impractical for numerical solutions. Since concrete compressive strain will limit the load-bearing capacity in all failure modes, the starting curvature can be 1% of the concrete’s tension cracking strain. The final curvature can be limited by the ultimate compressive strain of concrete, with the neutral axis depth assumed to be half of the section height, including the cover.

(1) $$\kappa =\frac{1}{\rho }=\frac{M}{E*I_{eff}}=\frac{\varepsilon_{cc}}{c}$$

(2) $$\varepsilon_{cc}=\kappa *c;\;\varepsilon_{ct}=\varepsilon_{f}=\varepsilon_{f,prestress}+\left(d-c\right)*\kappa ;\;\varepsilon_{st}=\kappa *\left(d-c\right);\;\varepsilon_{sc}=\kappa *(c-d^{\prime })$$

(3) $${f(z)}_{steel,\;concrete,\;frp}={\sigma (\varepsilon )}_{steel,\;concrete,\;frp}$$

(4) $$\int {f(z)}_{stee,\;concrete,\;frp}{*b*\;\partial }_{z}=F_{steel,concrete,frp}$$

(5) $$\sum Forces(z)=F_{cc}(z)+F_{sc}(z)-F_{ct}(z)-F_{st}(z)-F_{f}(z)$$

(6) $$c_{i+1}=c_{i}-\frac{\sum Forces\left(z=c_{i}\right)}{\partial \sum Forces\left(z=c_{i}\right)}\;\left|\sum Forces\left(c_{i}\right)\right|\;\leq 1\;N\;\;as\;convergence\;criteria$$

(7) $$M\left(z\right)=\sum \int F(z)*z*\;\partial_{z}$$

(8) $$P=\frac{M*4}{L}$$

(9) $$\delta =\frac{P*\;L^{3}}{48*E*I_{eff}}$$

The material constitutive law should be continuous, with no sudden breaks or steps. For concrete, the compressive stress function can be defined using several approaches available in the literature. This study uses Mander’s concrete compressive model [34] as it provides a continuous function and can be determined based solely on the material’s strength and elastic modulus. It is important to note that confinement is neglected in this study, as the shear details of the beam do not generate confinement stress sufficient to alter the compressive strength. Mander’s compressive model is given in Equation (10).

For concrete in tension, a linear elastic increase in stress occurs up to the cracking strain. After that, tensile strength decreases drastically. An exponential function is used to model this behavior, as shown in Equation (11). In this equation, parameters α and n are introduced to adjust the stiffness softening in tension, and they can be calibrated to represent the material’s behavior accurately.

The well-known elastic-plastic model is employed for the reinforcement, adjusting to smooth the transition from the linear elastic region to the yield plateau. Equation (12) is used for compressive and tensile rebar behavior, ensuring a continuous transition.

Being less complex, FRP material is modeled using a linear elastic relationship up to the failure strain, beyond which the stress drops to zero. The function in Equation (13) represents this. The failure strain is a critical parameter, as it can vary depending on the presence of an anchor and prestress. According to ACI 440, failure strain can be determined, with the anchor effect ignored in the calculation. However, if an anchor is present, ACI 440 suggests that the failure strain can be 90% of the failure strain for a non-anchored FRP.

**Figure 4:**
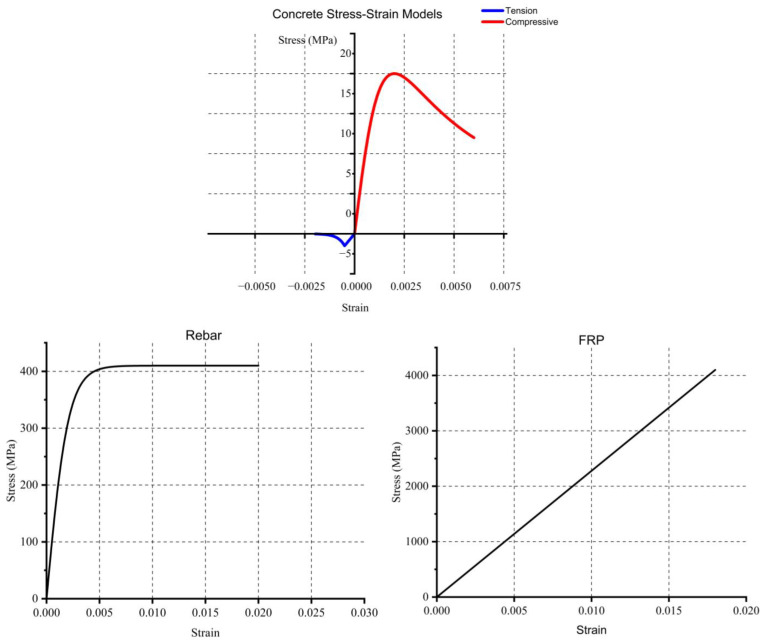
Material model in analytical study.

To illustrate the material model, the stress–strain curve for the materials is shown in Figure 4. Finally, to obtain the total load-displacement curve for all samples, a computer program is necessary. For this task, MATLAB (Version R2024a) [35] is chosen due to its powerful mathematical functions. The algorithm for the analytical method is provided in Appendix A.

(10) $$\sigma_{cc}\left(\varepsilon \right)=\frac{f_{cc}*x*r}{r-1+x^{r}},x=\frac{E_{c}}{\varepsilon_{co}},\;r=\frac{E_{c}}{E_{c}-E_{sec}},\;E_{c}=5000*\sqrt{f_{cc}}$$

(11) $$\sigma_{ct}\left(\varepsilon \right)=f_{ct}*\left(1-e^{-\alpha *\varepsilon_{ct}^{n}}\right),\;f_{ct}=0.35*\;\sqrt{f_{cc}}$$

(12) $$\sigma_{s}=f_{sy}*\tanh\left(\frac{E_{s}*\varepsilon_{s}}{f_{sy}}\right)$$

(13) $$\sigma_{f}=f_{t}*\exp\left(-\frac{\varepsilon_{f}}{\varepsilon_{ff}}\right)\;\varepsilon_{ff}=0.41*\sqrt{\frac{f_{cc}}{n*E_{f}}}\;\leq 0.9*\frac{f_{t}}{E_{f}}$$

## 4. Experimental and Analytical Results

**Figure 5:**
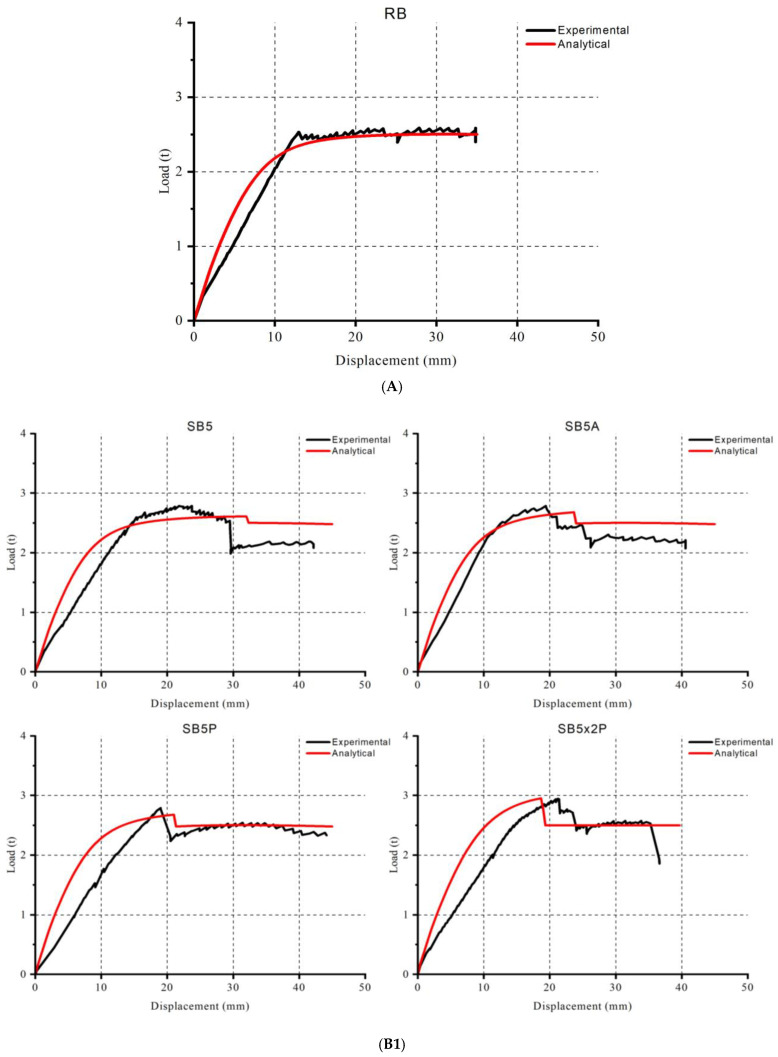

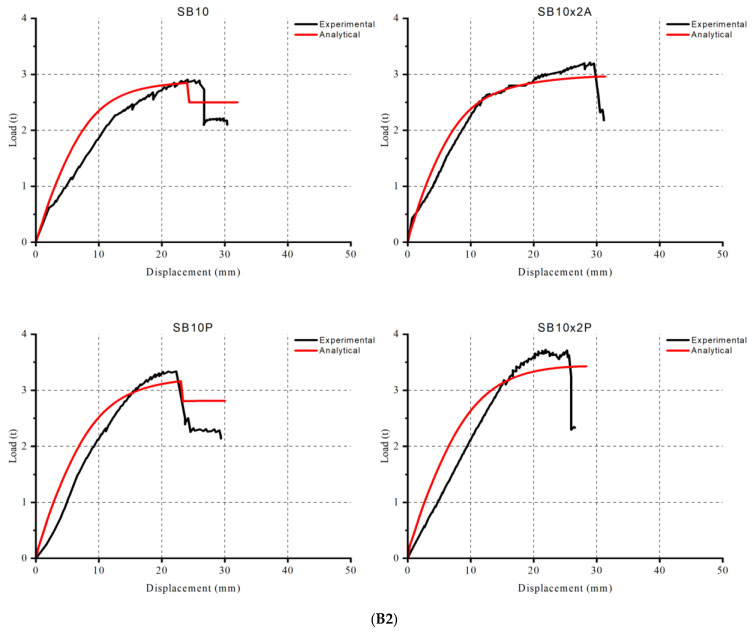
Experimental and analytical results. (**A**) Reference beam result. (**B1**) 50 mm strengthening results. (**B2**) 100 mm strengthening results.

**Figure 6:**
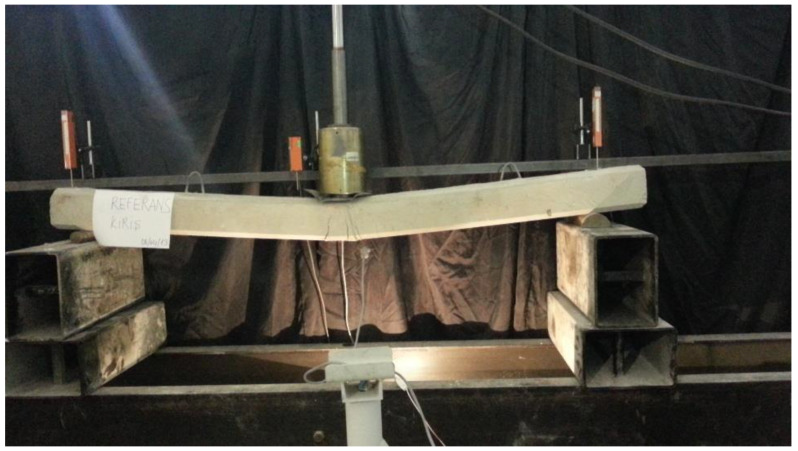
RB.

Load-displacement curves of experimental and analytical studies are given in Figure 5. According to the experimental results, the reference beam (RB) cracked at the low load level. It yielded upon reaching its ultimate load capacity and deformed until concrete crushing occurred, as shown in Figure 6. The strengthened beam SB5 failed after the debonding of the FRP strip, with residual ductile deformation. However, a significant drop in stiffness was observed post-debonding, indicating severe material damage. SB5A showed similar load-displacement behavior with a small increment in load-bearing capacity. Prestressing caused a few increments of the load-bearing capacity of SB5P, with FRP rupture occurring in the mid-stage of the load-displacement curve. The application of low-level prestressing did not result in an enhancement of the load-bearing capacity of the beam.

According to the experimental results, the reference beam (RB) cracked at the low load level. It yielded upon reaching its ultimate load capacity and deformed until concrete crushing occurred, as shown in Figure 6. The strengthened beam SB5 failed after the debonding of the FRP strip, with residual ductile deformation. However, a significant drop in stiffness was observed post-debonding, indicating severe material damage. SB5A showed similar load-displacement behavior with a small increment in load-bearing capacity. Prestressing caused a few increments of the load-bearing capacity of SB5P, with FRP rupture occurring in the mid-stage of the load-displacement curve. The application of low-level prestressing did not result in an enhancement of the load-bearing capacity of the beam.

**Figure 7:**
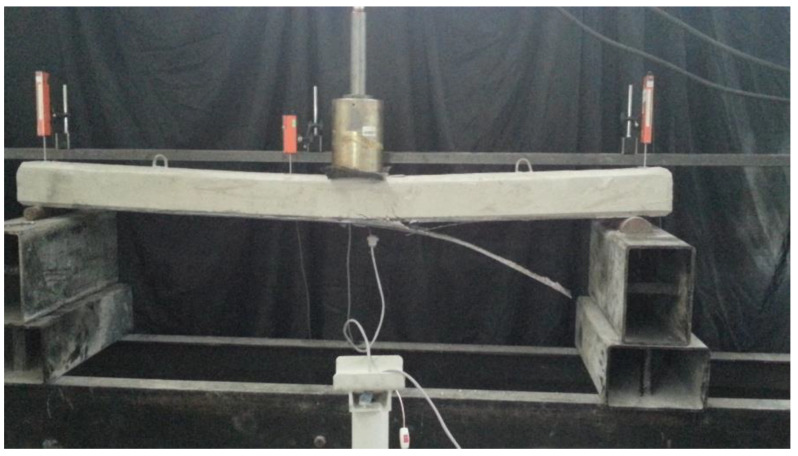
Debonding at SB10.

SB5x2P exhibited a significant increase in load capacity, reaching the highest capacity without stiffness reduction due to a higher FRP ratio and prestress. The FRP failure occurred in a mixed mode of rupture and intermediate crack debonding due to the severe damage in the concrete’s tension region. SB10 demonstrated the typical behavior of an FRP-strengthened RC beam, as shown in Figure 3. After the load reached 2.5 tons, the steel rebar reached yield stress, but the FRP maintained the beam’s ability to carry higher loads. The beam failed due to FRP debonding, resulting in a sudden collapse (Figure 7).

**Figure 8:**
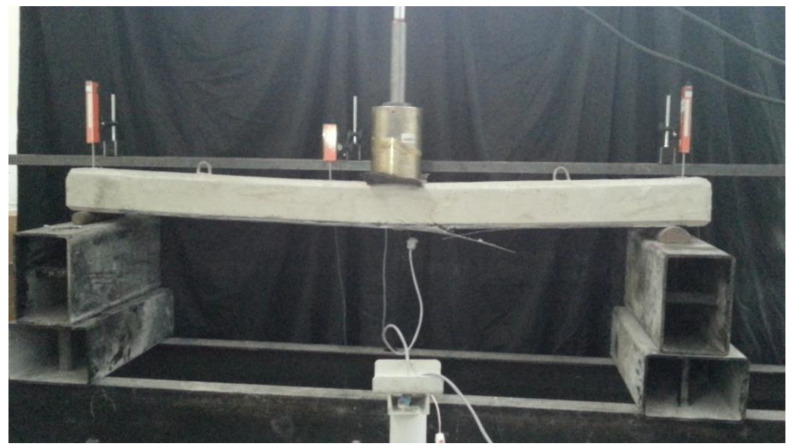
Rupture at SB10P.

In SB10x2A, the beam reached a higher load capacity than SB10. Like SB10, the rebar yielded before the FRP failed, and the beam collapsed due to FRP rupture. In SB10P (Figure 8), the beam reached nearly the same load level as the double-layer configuration. The beam sustained its stiffness up to the FRP rupture thanks to prestressing. Following the FRP failure, the beam collapsed catastrophically. Similar behavior was observed in SB10x2P, where the beam could sustain stiffness up to failure with minimal stiffness degradation. The beam collapsed after the FRP ruptured. Maximum load capacity was observed in SB10x2P.

The analytical approach successfully captured the total load-displacement curve, with a high correlation between the load capacity and displacement. Statistical analysis, including the Pearson coefficient, indicated strong similarity between the experimental and analytical curves. Most errors were observed in the cracked section stiffness and FRP debonding displacement. The loads at critical points, such as concrete tensile cracking, rebar yielding, and FRP debonding or rupture, were very close to those observed in the experimental results. However, the displacement at these load levels was slightly under-predicted. Comparing all samples, the most accurate results were obtained for RB, while the most significant discrepancies were found in the prestressed and double-layer configurations. The results are given in Table 2.

## 5. Discussion

Comparing the experimental results, it was found that a larger FRP application was more susceptible to delamination than a smaller one. However, preventing debonding was more challenging in the 50 mm FRP strengthening compared to the 100 mm width. This can be attributed to the difference in the interface area. The 50 mm strengthened samples were less successful in preventing rebar yielding than those with 100 mm strengthening, likely due to the difference in the FRP surface area. To mitigate this, it is possible to avoid premature failure through anchoring. Furthermore, when used as anchors, U-strips effectively controlled FRP debonding.

Another significant outcome of the study was the effect of the number of FRP layers. According to ACI 440, increasing the number of FRP layers typically decreases debonding strain, a trend supported by the experimental results. However, this effect did not hold for the prestressed samples. While prestressing led to premature debonding, the strain limit remained unchanged despite the increased FRP layers. The increased load-bearing capacity of the FRP laminates helped prevent debonding, but this was only true for samples with U-strip anchors.

Prestressing caused several changes in the behavior of the beams. Firstly, all prestressed samples exhibited an increase in beam stiffness. Additionally, load-bearing capacity improved, although ductility was reduced. Curvature and displacement were also limited, as shown in Figure 6 and Figure 7.

A comparison of experimental and analytical results revealed that the analytical approach successfully captured the total load-displacement curve, with some deviations. The primary deviations were observed in the cracked section stiffness. Several points are worth discussing:

- Concrete Compressive Stress: Mander’s model effectively captured the concrete compressive stress, accurately predicting beam failure in nearly all samples.
- Concrete Tension Model: While the concrete tension model successfully captured the cracking load, it did not sufficiently represent the cracked section stiffness. The model’s sharp decrease after cracking suggests that a simpler stiffness-softening model based on a stress–strain diagram is inadequate for capturing the more complex behavior of the material.
- Rebar Model: The exponential rebar model was generally successful in predicting yielding load and corresponding ductility. However, a yield delay occurred due to the exponential decay, indicating that a sharper model, such as the elastic-perfectly plastic model, would be preferable for better accuracy.
- FRP Model: The FRP model was generally accurate, as FRP behaves like an elastic-brittle material. The model effectively captured the limiting strain, which was crucial for predicting FRP debonding.

The highest deviation between experimental and analytical results was found in SB10x2P, which has the highest interaction area between FRP and concrete. Thus, the source of the error can be attributed to the concrete tension model and concrete–FRP interaction, including prestress in the analysis and composite assumption. Finally, the use of the easy prestressing machine (EPM) proved to be efficient. Even with a low amount of prestress, the overall behavior and load capacity of the beams changed. U-strips were particularly effective in applying prestress with the EPM, as no prestress loss was observed in the experiments. Both experimental and analytical results corroborated this outcome.

## 6. Conclusions

This study comprehensively investigated the use of prestressed FRP for strengthening RC beams through experimental and analytical methods. Nine samples were cast, and various strengthening configurations were tested, including different FRP widths, number of layers, anchoring techniques, and prestress levels. Several key outcomes were observed from the findings.

First, a larger FRP width delays debonding due to the increased interface area. Second, U-strip anchoring and prestressing successfully delay debonding, significantly mitigating debonding strain. It was concluded that anchoring and prestressing are essential for fully exploiting the benefits of FRP, as beams collapsed only after FRP rupture, highlighting their role in enhancing the overall performance of the beam with a 45% capacity increment.

Another key finding was the residual deformation capacity after FRP failure. When comparing the FRP area in terms of the number of layers and width, beams with smaller FRP strengthening exhibited a significant deformation reserve. However, this is only valid for under-reinforced RC beams. Thus, in the case of under-reinforced RC elements, the strengthening ratio must be carefully controlled to ensure high ductility, especially if debonding is effectively prevented.

The analytical studies showed a high correlation with the experimental results, with the maximum difference being only 8.5%, observed in the double-layer prestressed samples. The analytical approach consistently underestimated the load capacities of all samples, with most errors occurring in the cracked section stiffness, even though most of the samples showed a 90% or higher Pearson coefficient.

The analytical method, material model, and full composite assumption successfully predicted the beam behavior, but partial composite action was mostly observed in the samples lacking anchoring and prestressing. These samples failed under brittle conditions due to premature debonding. The study found that accurately capturing cracked stiffness requires incorporating partial composite action. Although the effective inertia was calculated using the general beam theory and analytical model, this was insufficient to predict cracked stiffness. This limitation arises from the partial stress distribution in the cracked zone, which the model did not fully account for.

The linear strain distribution was successful for concrete in compression, rebar, and FRP. Future research will involve the development of a hybrid method to improve the accuracy of cracked section stiffness prediction.

Future studies should also focus on expanding the easy prestressing machine (EPM) use for near-surface mounted FRP applications, in-groove FRP configurations, and other strengthening techniques with varied anchoring methods. These advancements will contribute to refining the understanding of FRP strengthening and provide a more comprehensive approach to enhancing the performance of RC beams.

## Figures and Tables

**Table 1:** Strengthening the configuration of samples.

Abbreviation of Sample	Width of FRP	Number of Layers	Anchoring	Prestressing
RB	-	-	-	-
SB5	50 mm	Single	-	-
SB5x2A	50 mm	Double	U-strip	-
SB5P	50 mm	Single	U-strip	0.001 mm/mm
SB5x2P	50 mm	Double	U-strip	0.001 mm/mm
SB10	100 mm	Single	-	-
SB10x2A	100 mm	Double	U-strip	-
SB10P	100 mm	Single	U-strip	0.001 mm/mm
SB10x2P	100 mm	Double	U-strip	0.001 mm/mm

**Table 2:** Comparisons of the results.

Specimen	Ultimate Load (Experimental) (t)	Ultimate Load (Analytical) (t)	Difference (%)	Pearson Coefficient
RB	2.59	2.5	3.5	0.99
SB5	2.78	2.61	6	0.91
SB5A	2.79	2.68	4	0.95
SB5P	2.79	2.68	4	0.82
SB5x2P	2.94	2.94	0	0.87
SB10	2.9	2.85	2	0.9
SB10x2A	2.96	3.21	7.8	0.89
SB10P	3.33	3.16	5.1	0.91
SB10x2P	3.75	3.43	8.5	0.91

**Figure A1:**
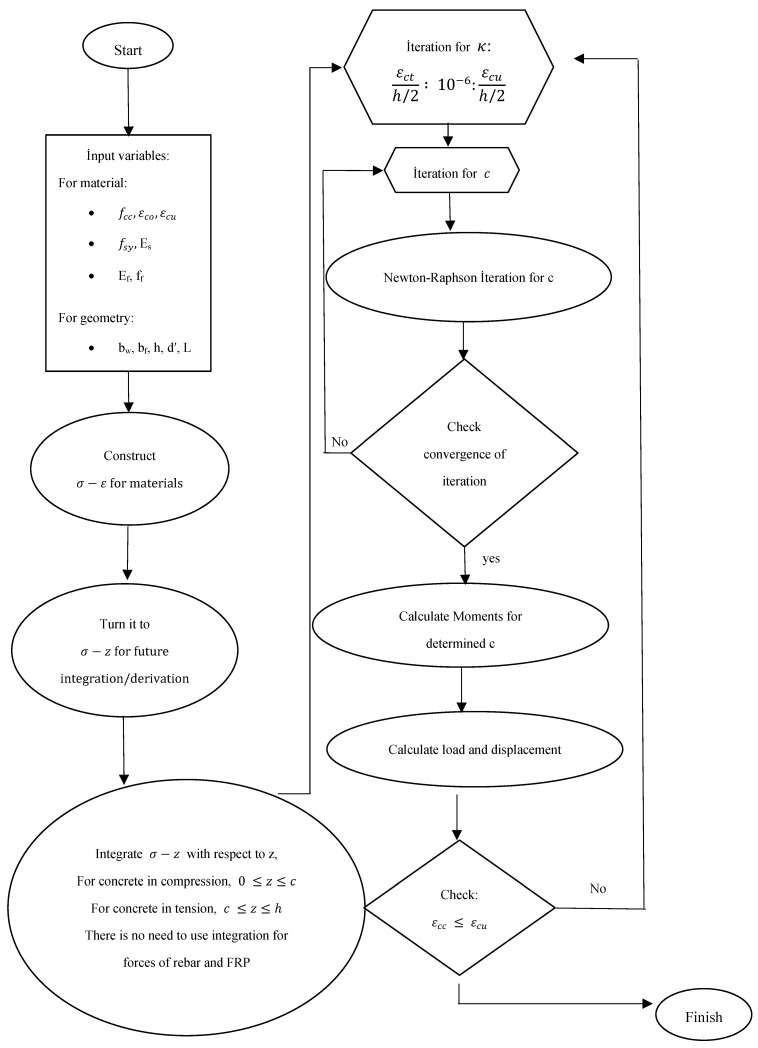
Algorithm to Construct Load-Displacement Curve.

## Data Availability

The data presented in this study are available on request from the corresponding author. The data are not publicly available due to planning to use our raw material in further studies.

## Nomenclature

P	load applied on the beam
δ	midspan displacement of the beam
L	span length
c	neutral axis depth
d′	cover
H	beam section height
b_f_	width of FRP layer
b_w_	beam section width
$\varepsilon_{cc}$	concrete compressive strain
$\varepsilon_{sc}$	rebar compressive strain
$\varepsilon_{st}$	rebar tensile strain
$\varepsilon_{ct}$	concrete tensile strain
$\varepsilon_{f}$	frp strain
$\sigma_{cc}\left(\varepsilon \right)$	concrete compressive stress
$\sigma_{s}\left(\varepsilon \right)$	rebar stress
$\sigma_{ct}\left(\varepsilon \right)$	concrete tensile stress
$\sigma_{f}\left(\varepsilon \right)$	FRP stress
M	moment
κ	curvature
ρ	curvature radius
E	elastic modulus
$I_{eff}$	effective moment of inertia
d	effective depth
$\varepsilon_{f,prestress}$	applied strain for prestressing
f(z)	transformed stress function of materials
$F_{cc}(z)$	force of concrete in compressive zone
$F_{sc}\left(z\right)$	force of rebar in compressive zone
$F_{ct}\left(z\right)$	force of concrete in tension zone
$F_{st}\left(z\right)$	force of rebar in tension zone
$F_{f}\left(z\right)$	force of FRP
$c_{i}$	neutral axis depth of i. iteration
x	coefficient of Mander model depending on E and $\varepsilon_{co}$
r	coefficient of Mander model depending on E and $E_{sec}$
$\varepsilon_{co}$	strain of concrete characteristic strength
$\varepsilon_{cu}$	ultimate strain of concrete in compression
$E_{sec}$	secant modulus of concrete for inelastic region
$f_{cc}$	concrete characteristic compressive strength
$f_{ct}$	concrete characteristic tensile strength
$f_{sy}$	rebar yielding strength
$\varepsilon_{ff}$	FRP ultimate strain
n	number of FRP strip
F_a_	force of analytical result
F_e_	force of experimental result
